# VEXAS syndrome in myelodysplastic syndrome with autoimmune disorder

**DOI:** 10.1186/s40164-021-00217-2

**Published:** 2021-03-19

**Authors:** Huijun Huang, Wenjun Zhang, Wenyu Cai, Jinqin Liu, Huijun Wang, Tiejun Qin, Zefeng Xu, Bing Li, Shiqiang Qu, Lijuan Pan, Gang Huang, Robert Peter Gale, Zhijian Xiao

**Affiliations:** 1 State Key Laboratory of Experimental Haematology, Institute of Haematology and Blood Diseases Hospital, Chinese Academy of Medical Sciences & Peking Union Medical College, Tianjin, China; 2 Hematologic Pathology Center, Institute of Hematology and Blood Diseases Hospital, Chinese Academy of Medical Sciences & Peking Union Medical College, Tianjin, China; 3 MDS and MPN Centre, Institute of Haematology and Blood Diseases Hospital, Chinese Academy of Medical Sciences, 288 Nanjing Road, Tianjin, 300020 China; 4 National Clinical Research Center for Blood Diseases, Institute of Hematology & Blood Diseases Hospital, Chinese Academy of Medical Sciences & Peking Union Medical College, Tianjin, China; 5 Divisions of Experimental Haematology and Cancer Biology, Cincinnati Children’s Hospital Medical Center, Cincinnati, OH USA; 6 Division of Experimental Medicine, Department of Medicine, Haematology Section, Imperial College London, London, UK

**Keywords:** Myelodysplastic syndromes, Autoimmune disorders, VEXAS syndrome, *UBA1* mutation, Cytoplasmic vacuolation

## Abstract

VEXAS (vacuoles, E1 enzyme, X-linked, autoinflammatory, somatic) syndrome is a newly-described adult-onset inflammatory syndrome characterized by vacuoles in myeloid and erythroid precursor cells and somatic mutations affecting methionine-41 (p.Met41) in *UBA1*. The VEXAS syndrome often overlaps with myelodysplastic syndromes (MDS) with autoimmune disorders (AD). By screening the *UBA1* gene sequences derived from MDS patients with AD from our center, we identified one patient with a p.Met41Leu missense mutation in *UBA1*, who should have been diagnosed as MDS comorbid with VEXAS syndrome. This patient respond poorly to immune suppressive drugs. Patients with MDS and AD who have characteristic vacuoles in myeloid and erythroid precursor cells should be screened for *UBA1* mutation, these patients are likely to have VEXAS syndrome and unlikely to improve with immunosuppressive drugs and should be considered for other alternative therapies.

**Supplementary Information:**

The online version contains supplementary material available at 10.1186/s40164-021-00217-2.

To the Editor,

VEXAS (vacuoles, E1 enzyme, X-linked, autoinflammatory, somatic) syndrome is a newly-described adult-onset inflammatory syndrome characterized by fevers, cytopenias, vacuoles in myeloid and erythroid precursor cells, dysplastic bone marrow, neutrophilic cutaneous and pulmonary infiltrates, nose and ear chondritis and vasculitis. The syndrome is associated with somatic mutations affecting methionine-41 (p.Met41) in *UBA1*, an X-chromosome gene encoding ubiquitin-like modifier-activating enzyme 1 [1]. Among the initial 25 patients reported with VEXAS syndrome, 6 met the World Health Organization (WHO) diagnostic criteria for myelodysplastic syndromes (MDS). In another study of 15 patients with VEXAS syndrome 5 had MDS [2]. These data indicate substantial overlap between VEXAS syndrome and MDS. Other data indicate an increased incidence of other autoimmune disorders (AD) in patients with hematological malignancies [3, 4], especially MDS [5–7]. In this study, we screened the *UBA1* gene sequences derived from MDS patients with confirmed AD from our center and identified one patient with a p.Met41Leu missense mutation in *UBA1*, who should have been diagnosed as MDS comorbid with VEXAS syndrome.

Five hundred and fourteen consecutive subjects with MDS from January 2013 to December 2019 were included in this study. Medical records were reviewed for evidence of an autoimmune disorder. Diagnosis of MDS was made according to 2016 revised WHO criteria [8]. Wright-Giemsa-stained blood and bone marrow slides were evaluated by two expert pathologists. Serum samples from 248 subjects (48%) were tested for cytokine levels. Blood samples from 216 (42%) subjects were assayed for lymphocyte sub-populations by multi-parameter flow cytometry (MPFC). Four hundred thirty-nine subjects (85%) with cytogenetics data were evaluated using the Revised International Prognostic Scoring System (IPSS-R) [9]. DNA from bone marrow mononuclear cells from 275 subjects (54%) underwent targeted 112-gene next generation sequencing (NGS) at diagnosis (Additional file 1: Table S1). All subjects provided informed consent in compliance with the Declaration of Helsinki.

Eighty-five subjects (16.5%) had an autoimmune disorder before their MDS diagnosis. The most common AD in MDS was rheumatoid arthritis (16, 18.8%), followed by psoriasis (14,16.5%), hypothyroidism (10,11.8%) and Behcet syndrome (9,10.6%) (Additional file 1: Table S2). Co-variates of all subjects are displayed in Table 1. Having an autoimmune disorder was associated with female gender (*p* = 0.024), higher frequency of trisomy 8 (*p* = 0.001) and a lower ratio of helper to suppressor T-cells (CD4/CD8 ratio; *p* = 0.032). Other co-variates were similar. Data from NGS showed no difference in mutation topography (Fig. 1a, b).

**Table 1:** Clinical and laboratory characteristics of MDS patients withand without autoimmune disorders (AD)

Characteristics	MDS with AD(n = 85)	MDS without AD(n = 429)	Total(n = 514)	P value
Sex(%)				0.024
Male	37(43.5)	245(57.1)	282(54.9)	
Female	48(56.5)	184(42.9)	232(45.1)	
Age,median(range),y	55(22–78)	57(7–84)	56(7–84)	0.538
WHO classification 2016(%)				0.719
MDS-SLD	3(3.5)	19(4.4)	22(4.3)	
MDS-SLD-RS	2(2.4)	16(3.7)	18(3.5)	
MDS-MLD	46(54.1)	224(52.2)	270(52.5)	
MDS-MLD-RS	4(4.7)	11(2.6)	15(2.9)	
MDS-EB1	17(20.0)	79(18.4)	96(18.7)	
MDS-EB2	9(10.6)	63(14.7)	72(14.0)	
MDS with isolated del(5q)	0	6(1.4)	6(1.2)	
MDS-U	4(4.7)	11(2.6)	15(2.9)	
Hb,median(range),g/L	79(41–155)	80(26–165)	80(26–165)	0.535
WBC,median(range), × 109/L	2.8(0.32–27.95)	2.78(0.41–25.45)	2.79(0.32–27.95)	0.786
ANC,median(range), × 109/L	1.14(0–6.52)	1.16(0–20.53)	1.16(0–20.53)	0.658
PLT,median(range), × 109/L	60(6–487)	62(2–603)	61(2–603)	0.912
BM blast,median(range),%	2.25(0–18)	2.5(0–19.5)	2.5(0–19.5)	0.618
CD4/CD8 T cell ratio	1.35(0.42–5.91)	1.67(0.37–8.42)	1.59(0.37–8.42)	0.032
Serum IL-6,pg/ml	6.22(2–31.5)	5.19(2–188)	5.28(2–188)	0.904
Serum TNF-α,pg/ml	21.6(4–1000)	14.9(4–478)	16.3(4–1000)	0.104
IPSS-R karyotype(%)(n = 439)				0.341
Very good	0	3(0.8)	3(0.7)	
Good	36(50)	216(58.9)	252(57.4)	
Intermediate	25(34.7)	86(23.4)	111(25.3)	
Poor	4(5.6)	19(5.2)	23(5.2)	
Very poor	7(9.7)	43(11.7)	50(11.4)	
Trisomy 8 positive(%)	22(30.6)	45(12.3)	67(15.3)	0.001
IPSS-R risk group(%)(n = 439)				0.558
Very low	0	12(3.3)	12(2.7)	
Low	21(29.2)	109(29.7)	130(29.6)	
Intermediate	27(37.5)	114(31.1)	141(32.1)	
High	15(20.8)	77(21.0)	92(21.0)	
Very high	9(12.5)	55(15.0)	64(14.6)	

MDS-SLD, MDS with single lineage dysplasia; MDS-RS-SLD, MDS with ring sideroblasts with single lineage dysplasia; MDS-MLD, MDS with multilineage dysplasia; MDS-RS-MLD, MDS with ring sideroblasts with multilineage dysplasia; MDS-EB1, MDS with excess blasts-1; MDS-EB2, MDS with excess blasts-2; MDS-U, MDS unclassifiable; Hb, haemoglobin; WBC, white blood count; ANC, absolute neutrophil count; PLT, platelet count; BM, bone marrow; IL-6, interleukin-6; TNF-α, α-tumor necrosis factor; IPSS-R, Revised International Prognostic Scoring System

**Fig. 1:**
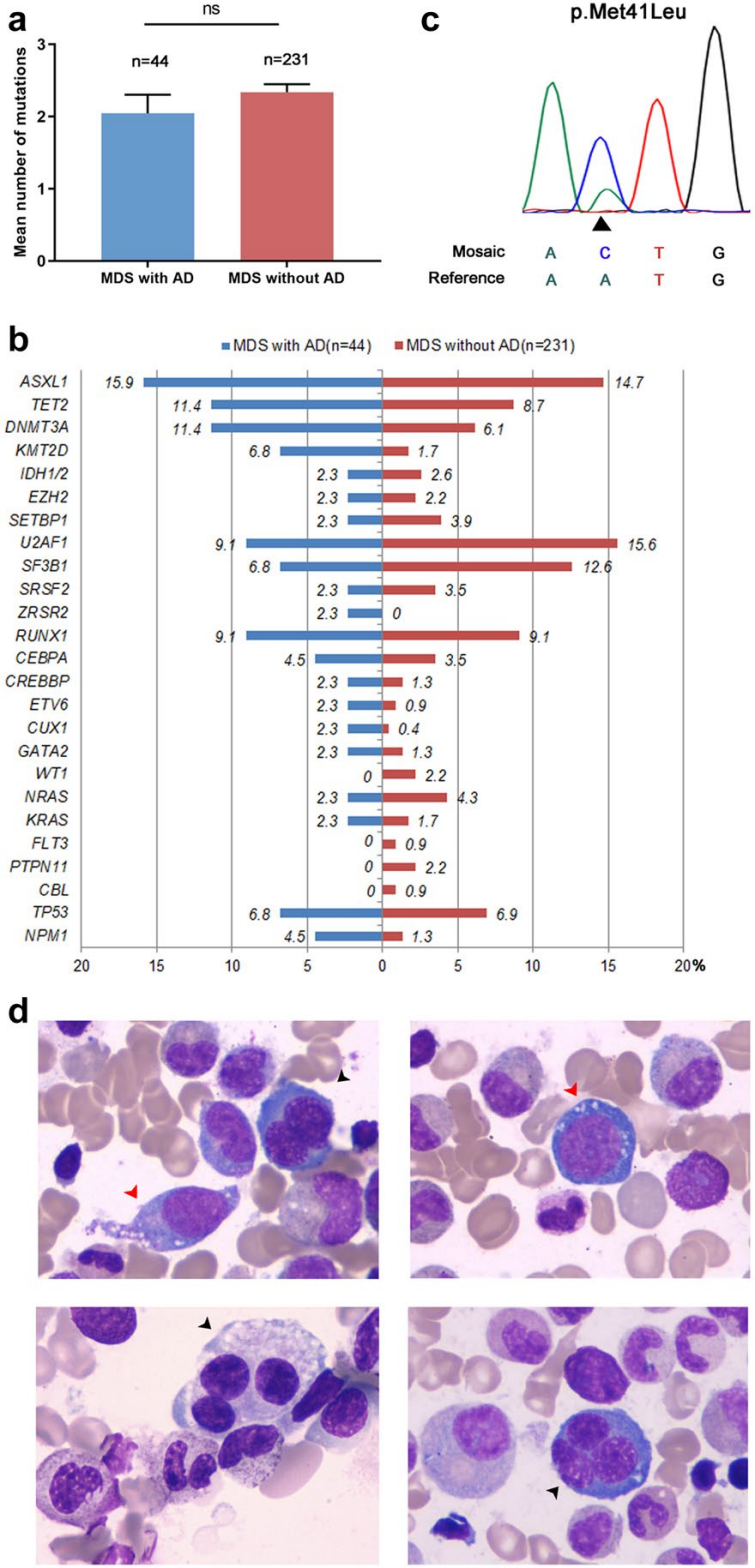
The mutational profile of myelodysplastic syndromes (MDS) patients with autoimmune disorders (AD) and the mutant site and bone marrow aspirate smears of the patient who had a *UBA1* mutation.**a** Mean numbers of gene mutations (showed by mean and SD) and** b** mutational profile in MDS patients with and without AD. **c** Chromatograms of *UBA1* somatic variant detected in one MDS patient.** d** The representative bone marrow aspirate smears of the patient with *UBA1* mutation showing dysplasia and characteristic cytoplasmic vacuolation of myeloid and erythroid precursors. The black arrows point to common dysplasia including nuclear abnormalities of erythroid precursor cells and the multinucleated micro-megakaryocyte. The red arrows point to cytoplasmic vacuolation of myeloid and erythroid precursors

Sanger sequencing for variants of interest in *UBA1* were performed in 47 patients who were diagnosed with AD and had available bone marrow DNA samples. Genomic DNA were extracted using TIANamp Genomic DNA Kit (TIANGEN DP304) according to the manufacturer’s instruction. PCR and Sequencing primers (For 5′-TCACCTCTGACCTTTTTTTC-3′, Rev 5′-ATGTTCTTAGCGATCTCCAC-3′) were designed using Primer Premier 5.0 software (Premier Biosoft International, Palo Alto, CA, USA). *UBA1* p.Met41 somatic mutations were detected by PCR performed with 2 × Hieff Robust PCR Master Mix (YEASEN 10106ES03). The PCR amplicons were then purified and Sanger sequenced using the ABI 3730XL (Applied Biosystems). Sequencing data were analyzed using Chromas (version 2.6.5, Technelysium Pty Ltd, USA) and all chromatograms shown are derived from Chromas.

A somatic variant in *UBA1* codon 41 (p.Met41Leu [NM_003334.3:c.121A → C]) (Fig. 1c) was detected in a 61-year-old male with rheumatoid arthritis diagnosed in local hospital 6 years ago and was seen by us because of cytopenias and macrocytic anaemia. The full blood count showed a hemoglobin of 67 g/L, MCV of 127.4 fL, WBC of 4.21 × 10^9^/L and platelet of 77 × 10^9^/L. A bone marrow aspirate showed hyper-cellularity with increases in all lineages without excess blasts with prominent dysplasia including pseudo-Pelger–Huët anomaly in neutrophils, megaloblastoid change and nuclear abnormalities in erythroid precursor cells and micro-megakaryocytes (Fig. 1d). There were no ringed sideroblasts. Typical cytoplasmic vacuolation of VEXAS syndrome were seen in myeloid and erythroid precursor cells (Fig. 1d). The karyotype was 46, XY[20]. No other somatic mutation was detected in NGS. He had an increased blood concentration of interleukin-6 (IL-6; 14.5 pg/mL) and α-tumor necrosis factor (TNF-α; 9.56 pg/ml).

The subject was diagnosed with MDS with multi-lineage dysplasia (MDS-MLD) with low risk group in IPSS-R. He received cyclosporine, danazol, thalidomide, low-dose prednisolone (below 10 mg/day) and RBC-transfusions. There was no haematologic improvement at 1 year and he remained RBC-transfusion-dependent at last follow-up on February 5, 2021.

Cytoplasmic vacuolation in hematopoietic cells is a non-specific abnormality seen in several diseases including MDS, sideroblastic anaemia, copper deficiency, zinc excess and acute alcohol exposure [10–13]. Different mechanisms of formation result in vacuoles with diverse histological features and distributions. One study reported vacuoles in patients with MDS are often of irregular shape with indistinct outlines and a tendency to coalesce suggesting the presence of glycogen [10]. In sideroblastic anaemia, cytoplasmic vacuoles occur predominantly in erythroid precursors. Copper deficiency can present as vacuolization of early erythroid and granulocytic precursors, but often accompanied by other morphological abnormalities, including prominent iron-containing plasma cells, ringed sideroblasts, and megaloblastic changes mimicking MDS [11]. In addition, a medical history that may cause copper deficiency, such as total parenteral hyperalimentation, is suggestive to diagnosis. In contrast, in VEXAS syndrome, numerous round vacuoles consisting of lipid droplets and disordered cellular organelles occur in myeloid and erythroid precursors [1, 2, 14].

Mutations affecting p.Met41 result in loss of the canonical cytoplasmic isoform of *UBA1* leading to decreased ubiquitylation, activated innate immune pathways and systemic inflammation. In one series no subject with VEXAS syndrome responded to disease-modifying anti-inflammatory drugs, while all were high-dose glucocorticoids-dependent [1]. In this series 10 of 25 subjects died from disease-related causes such as progressive anemia or therapy-related complications. In another study subjects with MDS and systemic inflammatory and autoimmune disorders were often steroid dependent [7]. Our patient concomitant with VEXAS syndrome also had poor hematologic response to immunosuppressive drugs and low-dose prednisolone.

In conclusion, patients with pan autoimmune disorders, especially those with typical VEXAS-related cytoplasmic vacuoles, should be tested for *UBA1* mutation. MDS patients with *UBA1* p.Met41 mutation probably have a poor prognosis regardless of a low IPSS-R score, and are unlikely to improve with immunosuppressive or hypo-methylating drugs and should be considered for alternative therapies including participation in clinical trials.

## Supplementary Information

**Additional file 1**:** Table S1**. Gene list of the 112-gene panel.** Table S2**. Frequency of autoimmune disorders identified in patients with MDS.

## Data Availability

The single institute data from this study is available from the corresponding author upon reasonable request.
