# Supplementary material for: VEXAS syndrome in myelodysplastic syndrome with autoimmune disorder
Source: Exp Hematol Oncol. 2021 Mar 19;10:23. doi: 10.1186/s40164-021-00217-2 (PMC7976711; doi:10.1186/s40164-021-00217-2)
Supplement: Supplementary file 1 — Additional file 1: Table S1. Gene list of the 112-gene panel. Table S2. Frequency of autoimmune disorders identified in patients with MDS. [file 40164_2021_217_MOESM1_ESM.docx]

**Supplementary information**

**Supplementary Table 1. Gene list of the 112-gene panel.**

| ABCB1 | ABL1 | ADAMTS13 | AKT1 | ALAS2 | ARID1A | ASXL1 | ATM |
| --- | --- | --- | --- | --- | --- | --- | --- |
| BCL2 | BCL6 | BIRC3 | BRAF | CALR | CBL | CCND1 | CCND3 |
| CDKN1A | CEBPA | MAF | MYC | CREBBP | CRLF2 | CSF3R | CUX1 |
| CXCR4 | CYLD | DDX3X | DIS3 | DNM2 | DNMT3A | ECT2L | EED |
| EGFR | EP300 | EPHA7 | EZH2 | FAM46C | FANCA | FANCC | FANCG |
| FAT1 | FBXW7 | FGFR3 | FLT3 | GATA2 | GATA3 | IDH1 | IDH2 |
| IKZF1 | IL7R | ITK | JAK1 | JAK2 | JAK3 | KIT | KRAS |
| SH2B3 | LYST | MAFB | MAPK1 | MLL2 | MPL | MUM1 | MYD88 |
| MYH11 | NF1 | NOTCH1 | NOTCH2 | NPM1 | NRAS | PAX5 | PDGFRB |
| PHF6 | PIK3CA | PRDM1 | PRF1 | PRMT5 | PRPF40B | PTEN | PTPN11 |
| RAB27A | RB1 | RELN | RUNX1 | SAMHD1 | SETBP1 | SF1 | SF3A1 |
| SF3B1 | SH2D1A | SMC1A | SMC3 | SRSF2 | STX11 | STXBP2 | SUZ12 |
| TAL1 | TEL | TET2 | TNFAIP3 | TP53 | TRAF3 | U2AF1 | U2AF2 |
| UNC13D | WAS | WHSC1 | WT1 | XIAP | XPO1 | ZMYM3 | ZRSR2 |

**Supplementary Table 2. Frequency of autoimmune disorders identified in patients with MDS**

| **Autoimmune diseases** | **N(%)** |
| --- | --- |
| *Systemic/connective tissue* |  |
| Rheumatoid arthritis | 16(18.8) |
| Behcet syndrome | 9(10.6) |
| Sjögren syndrome | 6(7.1) |
| Ankylosing spondylitis | 4(4.7) |
| UCTD | 4(4.7) |
| Systemic lupus erythematosus | 3(3.5) |
| Systemic sclerosis | 1(1.2) |
| Dermatomyositis/polymyositis | 1(1.2) |
| IgG4-related disease | 1(1.2) |
| *Blood* |  |
| Autoimmune haemolytic anaemia | 2(2.4) |
| *Cardiovascular* |  |
| Chronic rheumatic heart disease | 3(3.5) |
| *Endocrine* |  |
| Hypothyroidism | 10(11.8) |
| Hashimoto's thyroiditis | 2(2.4) |
| Graves' disease | 2(2.4) |
| *Skin* |  |
| Psoriasis | 14(16.5) |
| Cutaneous leucocytoclastic vasculitis | 3(3.5) |
| Gangrenous pyoderma | 2(2.4) |
| *Alimentary system* |  |
| Crohn’s disease | 3(3.5) |
| Ulcerative colitis | 2(2.4) |
| Autoimmune hepatitis | 1(1.2) |
| Primary Biliary Cirrhosis | 1(1.2) |

UCTD: Undifferentiated connective tissue disease
